# Effect of infrared drying on chemical and microbial properties of Cold‐Hardy grape pomace (Edelweiss and Marquette)

**DOI:** 10.1002/fsn3.3215

**Published:** 2023-01-06

**Authors:** Zeinab Mohammadi Shad, Chandrasekar Venkitasamy, Emily Kuelbs, Lucas Buren, Aude A. Watrelot

**Affiliations:** ^1^ Department of Food Science and Human Nutrition Iowa State University Ames Iowa USA; ^2^ Animal Health and Food Safety Services Division California Department of Food and Agriculture Sacramento California USA

**Keywords:** antioxidant activity, cold‐hardy grape pomace, Edelweiss cultivar, hot air drying, Marquette cultivar, phenolic content

## Abstract

This study aims to add value to a common wine industry waste by preserving bioactive compounds in cold‐hardy grape pomace (GP) and preventing any microbial growth under the proper drying conditions. Effects of infrared (IR) and hot air (HA) drying on the microbial and physicochemical properties such as color, phenolic compounds, and antioxidant activity of white (Edelweiss) and red (Marquette) GP were studied. The IR heating rapidly reduced the moisture content of GP from 55% wet basis (w.b.) to less than 10%, which resulted in a drying time reduction of 71.9% to 80.2% compared to HA drying. There were no significant differences in color parameters among the IR‐ and HA‐dried pomaces (*p* > .05). The phenolic content of ‘Edelweiss’ pomace was not significantly affected by both IR and HA drying, whereas the phenolic content of ‘Marquette’ pomace was substantially reduced from 274 mg/g dry extract in raw pomace to 127 mg/g dry extract after HA drying and to 141.9 mg/g dry extract after IR drying. Overall, the microbial load on the fresh pomace samples was dramatically reduced by IR heating, with a reduction of more than 99.9% when the pomaces were dried using IR at a temperature higher than 130°C. However, this high temperature of IR led to a significant reduction of DPPH antiradical scavenge activity for ‘Edelweiss’ pomace (*p* < .05). This study shows that using the IR approach, cold‐hardy ‘Edelweiss’ and ‘Marquette’ grape pomaces can be efficiently dried with the antioxidant activity maintained, which could be used in a variety of food products as a functional ingredient.

## Graphical abstract

In this study, we applied a novel drying technique on cold‐hardy grape pomace in order to add a value to this common wine industry waste. The drying technique was using infrared at various temperatures to identify the most appropriate drying condition to preserve phenolic compounds of grape pomace and reduce microbial activity. We evaluated the impact of those drying conditions on the color properties of the pomace, the phenolic compounds content, and the antioxidant activity of pomace extracts.

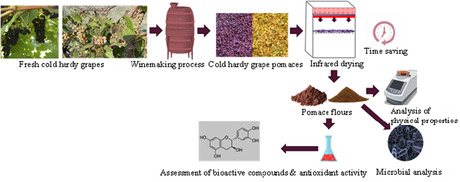

## 1 INTRODUCTION

Wine production in the U.S. accounts for 12% of global output, with more than 3 million liters of wine produced in 2017 (NAAW, 2021). About 20%–30% of the fresh weight of processed grapes is generated as grape pomace (GP), including stems, skins, pulp, and seeds (Beres et al., 2017). It is estimated that for every 6 L of wine produced, about 1 kg of GP is generated (Pereira et al., 2020) and wasted as it is commonly dumped in landfills.

On the other hand, the utilization of GP has become increasingly popular, as being rich in health‐beneficial compounds, including dietary fibers and phenolic compounds. Studies have shown various usage of GP, including as a growth medium for biomass production (Kingston et al., 2014), fortifying baked products (Nakov et al., 2020; Walker et al., 2014), chocolate formulation (Acan et al., 2021; Bursa et al., 2021), enrichment of dairy products, and salad dressing (Marchiani, Bertolino, Belviso, et al., 2016; Marchiani, Bertolino, Ghirardello, et al., 2016; Tseng & Zhao, 2013).

Phenolic compounds, such as tannins and anthocyanins which are antioxidant and anti‐inflammatory, reduce the risks of cardiovascular diseases and cancers (Unusan, 2020). During the red winemaking process after pressing, about 50% of tannins and between 15 and 60% of anthocyanins remained in the *Vitis vinifera* grape seeds and skins, that is, grape pomace (Ky & Teissedre, 2015). In the U.S. Midwest, cold‐hardy interspecific hybrid grape cultivars, resistant to harsh cold winter, are used to produce wines. There are differences in grape chemistry between the commonly grown *Vitis vinifera* grape varieties and cold‐hardy interspecific hybrids. It has been observed that in interspecific hybrid wines, fewer phenolic compounds are released in wines than in *Vitis vinifera* wines (Rice et al., 2017). Therefore, it has been suggested that those compounds are retained in the cold‐hardy grape pomace (Rice et al., 2017; Watrelot & Norton, 2020). Thus, there is a tremendous economic opportunity to valorize cold‐hardy GP and its compounds in the fields of health and nutraceuticals, as the consumers' demand for bio‐sourced products is increasing continuously (Fontana et al., 2013).

Grape pomace contains a significant amount of moisture that can negatively impact its quality during storage if not appropriately dried (Majerska et al., 2019). Conventional drying, such as hot air (HA) drying, is the most common method to reduce the moisture content (MC) below 14% to prevent microbial growth (El‐Mesery & Mwithiga, 2014). However, HA drying takes a long time (hours) to reduce the moisture content in the pomace, which promotes microbial development and results in nutritional and bioactive compound breakdown (Martynenko & Kudra, 2016). Establishing a rapid and effective drying approach is essential because of the lengthier drying times and losses in phenolic content associated with traditional drying processes. Infrared (IR) drying is an efficient heating technology that proposes several processing advantages, such as rapid drying and microbial inactivation, while improving the safety and quality of food products (Pan et al., 2019).

Hence, in the current study, the development of IR heating takes a further step as an efficient technology to dry cold‐hardy GP to a stable product that can prevent microbial growth. It is hypothesized that drying GP using IR heating will provide a sustainable green way to process postwinemaking waste, creating salubrious economic and environmental benefits.

Also, no research has been carried out yet on the impact and optimization of IR heating on cold‐hardy interspecific hybrid GP. The objectives of this study were (1) to evaluate the effectiveness of the IR heating in cold‐hardy GP valorization and optimize the IR drying parameters, (2) to determine the effects of the IR drying process on the phenolic content and antioxidant properties of cold‐hardy GP, and (3) on the microbial properties of the cold‐hardy GP.

## 2 MATERIALS AND METHODS

### 2.1 Grape pomace samples and preparation

In this study, two types of cold‐hardy GP were used: (1) pomace of white grape, cultivar Edelweiss, obtained after crushing and pressing, which was kindly supplied by the Cellar at White Oak Winery in September 2020; and (2) pomace of red grape, cultivar Marquette, obtained after alcoholic, malolactic fermentation and pressing from a research project carried out by Dr. Watrelot, Iowa State University (Ames, IA) in October 2020. The pomace samples were packed in sealed plastic tubs (68 L) and immediately stored in a freezing room (−20°C) at Iowa State University until further use. The pomace samples were thawed at room temperature (25°C) for 24 h before drying.

Stems were manually separated from both pomace samples to collect seeds and skins. Also, the pomace of the ‘Edelweiss’ grape contained rice husk, commonly used to increase juice yield during pressing. After the drying process, the husk was manually separated prior to the grinding, using five selected U.S. standard sieves (Nos. 6, 7, 8, 12, and 30) and a pan fitted into the last sieve. The depth and outer diameter of the sieves were 2.5 cm and 203 mm, respectively.

### 2.2 Infrared heating system

**FIGURE 1:**
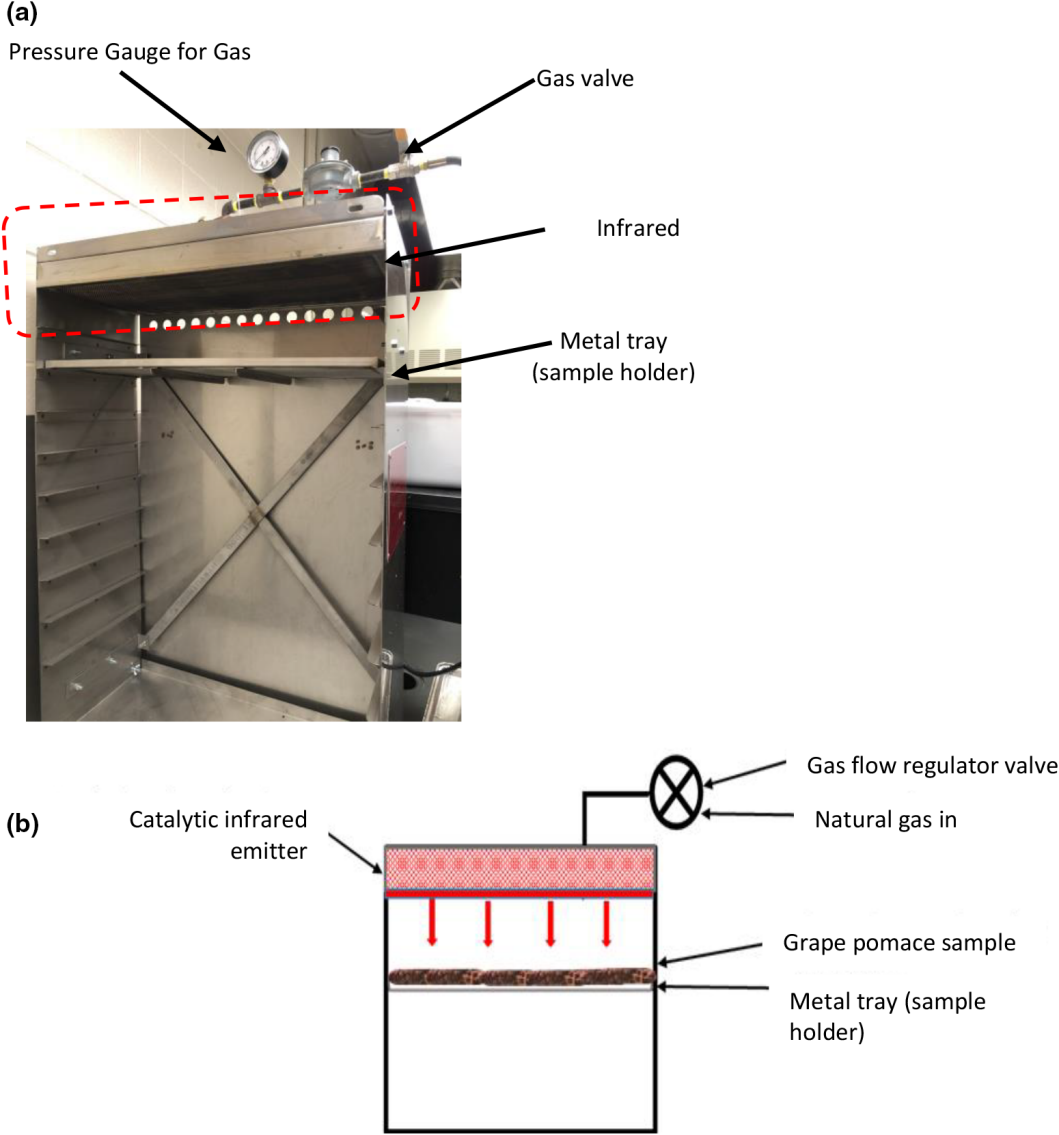
Infrared (IR) heating system (a); schematic of IR treatment of grape pomace (b).

A catalytic IR heating system (Catalytic Drying Technologies LLC), assembled in‐house in the Department of Food Science and Human Nutrition at Iowa State University, was used for this study. The bench‐top (30 cm by 61 cm) catalytic IR heater was powered by natural gas. The unit consists of a heating chamber, gas flow regulator, pressure gauge for gas, metal trays, and gas‐powered catalytic IR emitters (Figure 1).

Preliminary tests helped us to select three IR drying conditions in order to prevent GP from being burned. The IR treatments were performed at the following conditions of gas pressures (kPa) and product‐to‐emitter gap distances (cm): 0.2 kPa and 14.6 cm for IR‐low heating (IR‐LH), 0.5 kPa and two gap distances of 14.6 cm (first 8 min) and 22.2 cm (until MC < 10%) for IR‐modified heating (IR‐MH), and 0.3 kPa and 14.6 cm for IR‐high heating (IR‐HH). The product‐to‐emitter gap distance and gas pressure change allowed for the variable emitter and product surface temperature (Table 1).

**TABLE 1:** Experimental design of the infrared (IR) drying conditions used.

IR treatments	Abbreviation code	IR system configuration		
		Gas pressure (kPa)	Product to emitter gap distance (cm)	Emitter temperature (°C)
IR – low heating	IR‐LH	0.2	14.6	283
IR – modified heating	IR‐MH	0.5	D_1_ (14.6) D_2_ (22.2)	390
IR – high heating	IR‐HH	0.3	14.6	325

The emitter and pomace surface temperatures were monitored periodically using a handheld VWR traceable dual laser IR thermometer (VWR International LLC).

### 2.3 Experimental setup

The IR heating system was first preheated by using the electric heater for about 30 min. Then, the natural gas valve was opened and used to heat the emitter until the surface temperature of the IR emitter reached about 300–400°C. The electric power supply was disconnected after an additional 10–15 min after the gas had been introduced. Pomace samples (220 g) were spread out on nonstick aluminum dishes (40 cm long × 30 cm wide) in a single layer. When the GP sample reached about 40%–50% MC during IR and HA drying processes, the samples were ground and homogenized using a CapressoTM coffee grinder for 10 s. Then, the drying continued for the homogenized GP until the final MC reached less than 10%. During IR heating, the ground sample was mixed thoroughly to provide uniform heating.

As stated in Table 1, we devised three IR experimental setups capable of rapidly drying pomace without burning it; these parameters were determined through a series of trial tests. The IR‐low heating (IR‐LH) was run at a gas pressure of 0.2 kPa, and the product‐to‐emitter gap distance of 14.6 cm provided the low temperature of the product surface (71–78°C), which is IR treated. IR‐high heating (IR‐HH) was done at a gas pressure of 0.3 kPa, and the product‐to‐emitter gap distance of 14.6 cm provided a higher temperature of the product surface (131–141°C). For IR‐modified heating (IR‐MH), the IR heating system was adjusted at 0.5 kPa gas pressure. Next, the pomace samples were dried at two different product‐to‐emitter gap distances: drying at the gap distance of 14.6 cm to reach 40% MC, then increasing the gap distance to 22.2 cm, which provided a medium temperature of the product surface. The increased gap distance of 22.2 cm was used in order to avoid overheating or burning of the low‐moisture grape pomace at the final stages of drying.

For HA drying, the GP sample of 220 g was spread out on the same nonstick aluminum pan at a single layer and was dried with a HA dryer at 70°C. The sample was ground at 40%–50% MC and then drying was continued until MC was reduced to less than 10%.

The pomace weight loss over time was monitored intermittently throughout the HA and IR drying by taking out the sample and weighing using an electronic balance (Quintix 412‐1S; Sartorius) with a sensitivity of 0.01 g and placing back in the dryer very quickly. During IR and HA drying, the samples were weighed after every 10 min and 30 min, respectively. The initial MC and initial weight, as well as the pomace weight at time t, were used to calculate the pomace MC at time *t* during the drying process. The GP samples dried with IR were compared with those dried with the HA as a reference for the conventional drying method.

After IR and HA drying, the GP samples were ground to a particle size of less than 1 mm using a CapressoTM coffee grinder for 1 min. The dried and ground samples were placed in sealed plastic containers and stored in a freezer at −20°C until further analysis.

### 2.4 Moisture content analysis

The initial and final MC of pomace samples were determined using a forced air oven (Isotemp Oven model 655F; Fisher Scientific) set at 105°C until reaching consistent weight, and the percentage of weight loss was calculated as wet based (AOAC, 1997). The moisture reduction during drying periods was frequently monitored by measuring pomace weight loss throughout the drying process. All MCs are on a wet basis (w.b.) unless otherwise specified.

### 2.5 Color analysis

The color parameters, L* (lightness), a*(redness), and b* (yellowness) values of dried GP, were determined using a Hunter Lab ColorFlex EZ colorimeter (Hunter Associate Laboratory Inc.) on 500 mg of dried GP uniformly spread as a layer in a petri dish (60 × 15 mm).

### 2.6 Total phenolic and tannin contents

Phenolic compounds were extracted from raw pomace and dry ground pomace using a conventional technique, where 10 ml of 70% acetone containing 0.05% TFA was added to 500 mg of GP and flushed with nitrogen before shaking overnight in dark at room temperature. After filtration using a Buchner and paper filter Whatman™ grade 1, the solvents were evaporated using a heating block at 32°C with a nitrogen flush to avoid oxidation. The aqueous extracts were then frozen at −80°C and freeze‐dried. The amount of the extract was recorded.

After extraction, the total iron‐reactive phenolic content was determined using the Adams‐Harbertson assay (Heredia et al., 2006). Briefly, 5 g/L of dry extract in model wine (13% ethanol, 5 g/L tartaric acid, pH 3.5) was prepared, and 75 μl of this solution was placed in a 1 cm pathlength cuvette, and 800 μl of resuspension buffer (pH 9.4) was added. After vortexing and 10‐min incubation at room temperature, the absorbance was measured at 510 nm using a UV–Visible spectrophotometer (Genesys 150, Thermo Scientific). Next, 125 μl of ferric chloride solution was added, and 10 min after vortexing, the absorbance was read at 510 nm. The total iron‐reactive phenolic content was then expressed as mg (+)‐catechin equivalents per gram of GP extract.

The tannin content from the dry extracts was measured using the protein precipitation method with bovine serum albumin (Heredia et al., 2006). The same solution of dry extracts in model wine was used for this method, and the tannin content was determined and expressed as mg/g of dry extract as (+)‐catechin equivalent.

### 2.7 Antioxidant activity

For the antioxidant activity of the dry pomace extracts, a stable free radical α‐diphenyl‐β‐picrylhydrazyl (DPPH) was used, and the scavenging capacity of the dry pomace extracts was evaluated following a previously published method (Carmona‐Jiménez et al., 2014). Briefly, a stock solution of DPPH (2, 2‐diphenyl‐1‐picrylhydrazyl) at 2 mM in methanol was prepared fresh before the analysis and kept in the dark and stored in fridge under darker conditions before being used. Each sample was diluted in model wine at four different concentrations (0.5, 0.25, 0.1, and 0.05 g/L). Into each, 100 μl of the diluted sample, 800 μl of methanol, and 100 μl of the DPPH stock solution were added. A DPPH blank was prepared to contain 900 μl of methanol and 100 μl of DPPH stock solution. A blank of diluted samples was prepared to contain 900 μl of methanol and 100 μl of diluted samples. The absorbance readings were carried out against a blank of methanol. Each sample was vortexed and kept in the dark at room temperature for 30 min. After incubation, the absorbance values of the samples were taken using a UV–visible spectrophotometer (Genesys 150, Thermo Scientific) at 517 nm. The curves were then used to determine the concentration of pomace extract solution required to inhibit 50% of DPPH.

### 2.8 Microbiological analysis

The number of viable microorganisms for selected microbial groups, including aerobic plate count (APC), spore‐forming bacteria (SPO), and yeasts and molds (Y&M), were evaluated. Briefly, GP samples were taken aseptically from several areas of the same GP and combined to have 25 g sample. The 25 g GP sample was then homogenized in 225 ml of sterile diluent prior to analysis. The culture techniques and incubation procedures are shown in Table 2. After incubation, samples of the broth were streak plated on various mediums depending on the microorganisms. Coliform bacteria and Salmonella were not detected using the direct plating method described here. For all samples tested, the numbers of viable coliform bacteria or Salmonella were below the analytical method's detection limit (10 CFU/g = 1.0 Log CFU/g) and were, therefore, not shown.

**TABLE 2:** Details of microbial analytical procedures.

Description	Microbial group code	Culture technique^a^	Incubation conditions
Aerobic plate count	APC	Spread plate on TSAYE	35°C, 48 h
Spore‐forming bacteria (aerobic)	SPO	Heat shock of sample; spread plate on TSAYE	35°C, 48 h
Yeast and mold	Y&M	Spread plate on DRBC	25°C, 5 days

Abbreviations: APC, aerobic plate count; DRBC, Dichloran rose Bengal chloramphenicol agar; SPO, spore‐forming bacteria; TSAYE, Tryptic soy agar supplemented with 0.6% yeast extract; Y&M, Yeast and mold.

^a^ Detection limit = 10 (1.0 Log) Colony‐Forming Units (CFU) per gram of grape pomace.

### 2.9 Data analysis

All analyses were done in triplicate, and the results were reported as mean ± standard deviation (SD). Statistical results were significant when *p* < .05. A one‐way ANOVA was carried out on all the samples using a Tukey's test (LSD) with 95% confidence, using XLSTAT 2021 software.

## 3 RESULTS AND DISCUSSION

### 3.1 Moisture removal

The initial MCs of Edelweiss and Marquette pomace were reduced from 59.7 ± 3.6% and 50.1 ± 1.5%, respectively, to a final MC of less than 10%. The total drying rate of both GP samples using catalytic IR heat was faster than that of HA drying (Table 3). On average, 26 min of IR heating was needed to decrease the initial MC to less than 10% in ‘Edelweiss’ and ‘Marquette’ pomace samples, while HA drying required more than 90 min for the same moisture reduction. The fastest drying rate was achieved using IR‐MH, with an average drying duration of 16 min. However, about 45 min using IR‐LH was required to obtain the same MC reduction. For ‘Edelweiss’ pomace, the IR‐LH, IR‐MH, and IR‐HH significantly reduced the drying time by 51.6%, 85.2%, and 78.9%, respectively, compared to HA drying. Similarly, a higher reduction in drying time for ‘Marquette’ pomace was obtained by IR compared to HA drying (75.8%, 84.6%, and 81.3%, by IR‐LH, IR‐MH, and IR‐HH, respectively). Furthermore, GP dried faster under IR‐MH than under IR‐HH (Table 3). This can be due to the quick absorption of IR energy by the surface layer of GP, which increases the internal water vapor pressure and opening of pores and, thereby, a shorter drying time (Abano et al., 2014).

**TABLE 3:** Drying time and product surface temperature are impacted by drying method.

Pomace	Treatment	Time (min)^†^	Product surface temperature (°C)
Edelweiss	IR‐LH	62 ± 2.71^b^	71
	IR‐MH	19 ± 1.50^c^	124
	IR‐HH	27 ± 1.74^c^	131
	Hot air	128 ± 3.14^a^	—
Marquette	IR‐LH	22 ± 1.03^b^	78
	IR‐MH	14 ± 1.16^c^	125
	IR‐HH	17 ± 1.10^bc^	141
	Hot air	91 ± 2.25^a^	—

Abbreviations: IR‐HH, infrared high heating; IR‐LH, infrared low heating; IR‐MH, infrared‐modified heating.

^†^ The different letters for each grape pomace indicate the statistically significant differences between the drying treatments (*p* ≤ .05).

In general, other researchers have offered IR heating as an efficacious drying approach to conquer the deficiencies of conventional drying methods. For example, IR heating at the 20‐cm gap distance showed the highest drying rate while reducing the drying time of GP by 47.3% compared to convective, sequential IR, and convective drying (Sui et al., 2014). Similarly, IR heating of seeded black GP resulted in uniform temperature distribution, high energy, and energy efficiencies, with IR at 50°C as the most suitable drying temperature (Dolgun et al., 2020). The IR heating has also shown remarkable results in processing other crops, such as corn drying and disinfection (Mohammadi Shad et al., 2021; Wilson et al., 2021) as well as berry and brewery‐spent grain drying (Mchugh et al., 2020; Zhang et al., 2019).

### 3.2 Color

The L*, a*, and b* parameters of ‘Edelweiss’ and ‘Marquette’ pomaces dried with IR and HA are shown in Table 4. The average values of L*, a*, and b* parameters for ‘Edelweiss’ pomace were 39.9, 10.7, and 23.0 when dried with IR and were 44.0, 10.7, and 26.1 when dried with HA, respectively. The lightness (L*) was not significantly different in ‘Edelweiss’ pomaces dried with HA, and IR under three heating conditions. The corresponding parameters for ‘Marquette’ pomace were 30.4, 8.9, and 8.2 when dried with IR and were 30.7, 9.5, and 7.0 when dried with HA, respectively. Moreover, the temperature of IR heating (LH, MH, and HH) did not significantly affect the color of dried pomaces (Table 4). The results agreed with previously published data as IR did not significantly affect the color of the dried product, sweet red peppers, compared to conventional air drying (Guclu et al., 2021). Convective thermal drying has shown a significant impact on the color of treated GP. Air drying significantly affected the L* and a* parameters of GP because of intensive browning due to thermal oxidation compared to nonthermal electrohydrodynamic drying (Martynenko & Kudra, 2016).

**TABLE 4:** Color parameters (L*, a*, and b*) of grape pomace samples as influenced by drying method.

	Drying method	L*	a*	b*
Edelweiss	IR‐LH	40.68 ± 1.70	10.81 ± 0.23	22.67 ± 1.05
	IR‐MH	38.73 ± 2.50	11.08 ± 0.28	24.39 ± 2.62
	IR‐HH	40.16 ± 1.58	10.33 ± 0.75	22.08 ± 1.23
	Hot air	44.04 ± 0.16	10.69 ± 0.06	26.09 ± 0.56
Marquette	IR‐LH	31.18 ± 1.85	9.10 ± 0.20	7.86 ± 0.44
	IR‐MH	29.70 ± 0.13	8.93 ± 0.25	8.60 ± 0.29
	IR‐HH	30.18 ± 0.25	8.71 ± 0.28	8.22 ± 0.46
	Hot air	30.06 ± 0.68	9.52 ± 0.15	7.02 ± 0.57

Abbreviations: IR‐HH, infrared high heating; IR‐LH, infrared low heating; IR‐MH, infrared‐modified heating.

### 3.3 Phenolic and tannin contents

**FIGURE 2:**
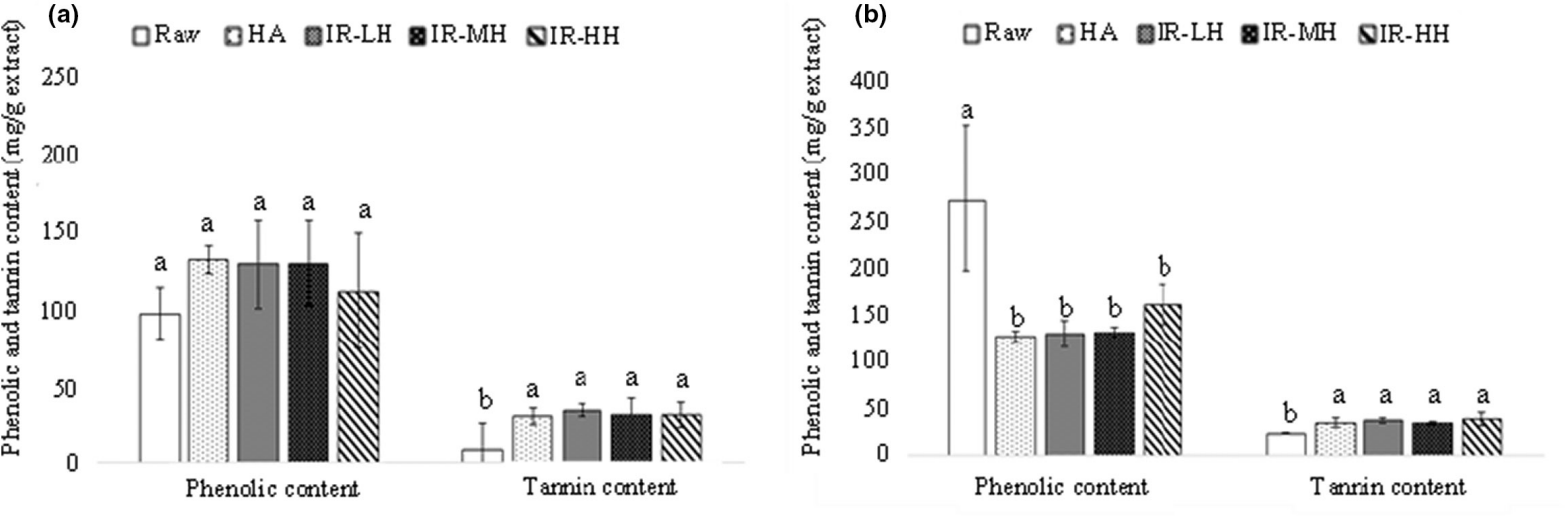
Phenolic and tannin contents (mg/g dry extract) of Edelweiss (a) and Marquette (b) dry pomace extracts. HA, Hot air drying; IR‐HH, Infrared high heating; IR‐LH, Infrared low heating; IR‐MH, Infrared modified heating.

The phenolic and tannin contents of raw and dried GP samples are shown in Figure 2. The phenolic and tannin contents in raw ‘Edelweiss’ pomace (white GP) were 97.2 and 8.6 mg/g dry extract as (+)‐catechin equivalent, respectively, and were 273.7 and 23.2 mg/g dry extract as (+)‐catechin equivalent, respectively, in raw ‘Marquette’ pomace (red GP). Similarly, Xu et al. (2015) also reported a higher average phenolic content in red GP (122.9 mg Gallic Acid Equivalent (GAE)/g extract) than white GP (77.3 mg GAE/g extract), as red GPs contain similar phenolic compounds than in white GPs plus anthocyanins and pigmented tannins. The higher content of phenolic compounds in red GP also explains the lower color parameters a* and b* than in white GP as anthocyanins and pigmented tannins are responsible for the dark red color. Both drying methods used in the current study, IR (all three conditions) and HA, did not affect the phenolic content in ‘Edelweiss’ pomace (Figure 2a) but significantly reduced it in ‘Marquette’ pomace (Figure 2b). Also, more tannin contents were found for dried ‘Marquette’ and ‘Edelweiss’ pomaces than fresh, raw pomaces. There was no significant difference in phenolic and tannin contents between the HA‐ and IR‐dried GP samples. The average tannin content in raw, IR‐dried, and HA‐dried pomaces of ‘Edelweiss’ was about 8.6, 29.8, and 31.8 mg/g dry extract as (+)‐catechin equivalent, respectively, whereas these contents were 23.2, 34.5, and 36.2 mg/g dry extract as (+)‐catechin equivalent in ‘Marquette’ pomace, respectively. During the white winemaking process, grapes are pressed to get the pomace and juice and then go through alcoholic fermentation. This process is different from red winemaking, in which grapes go through alcoholic fermentation to extract anthocyanins and tannins and then are pressed to obtain the wine (rich in tannins) and the pomace. Therefore, it was expected that tannins were released in red wine, and a very low content would be in the red GP. The tannin content in ‘Edelweiss’ GP was lower than in ‘Marquette’ GP. This result was in agreement with the hypothesis that the low tannin content in interspecific hybrid red wines might be due to a higher retention of those compounds in the cell wall matrix, including proteins and polysaccharides (Springer & Sacks, 2014; Watrelot, 2021).

The drying process can explain the higher phenolics content in dried GP obtained in the current study as it can break and disrupt cell walls, providing large intercellular spaces to extract cellular substances better (Drosou et al., 2015). However, researchers have reported different temperature impacts on phenolic compounds and tannin content. Similar to our study, the thermal treatment of grape seed extract and GP in an electric furnace at 100°C did not change the total extractable polyphenol and tannin contents after 15, 30, and 60 min (Chamorro et al., 2011). However, it was found that the total phenolics content of GP dried in a climatic chamber at 40°C and 10% relative humidity was significantly higher (three times or more) than that of fresh GP due to extractability improvement of phenolic compounds (Carmona‐Jiménez et al., 2018). On the contrary, another study indicated that higher drying temperatures caused a higher loss of phenolic compounds as the phenolic content of raw GP (165 mg GAE/g) decreased by 87.0% and 95.4% after drying at 60°C for 26 h and at 85°C for 5 h, respectively (Goula et al., 2016). Room temperature and vacuum oven drying at 70°C also resulted in a significant loss of phenolic compounds because of a longer drying time and thus higher anthocyanin degradation (Yu, 2014). In another study, conventional drying at 40°C, 55°C, and 70°C for 2 h resulted in a significant reduction of bioactive compounds and antioxidant activity compared with intermittent drying followed by 5 and 10 min of tempering. The GPs dried using intermittent drying at 40°C for 5 min showed the lowest loss of bioactive compounds, including 20.6% and 14.5% for total phenolic compounds and total flavonoids, respectively (Borges et al., 2020). There was also more than a 30% reduction of bioactive compounds when two red wine grape pomaces of Pinot Noir and Merlot were dried using 40°C conventional and vacuum drying or ambient air drying (25°C) compared to freeze drying (Tseng & Zhao, 2012). In the current study, IR drying was found to be the practical drying method to preserve the phenolic compounds of ‘Edelweiss’ pomace but led to a reduction of phenolics content in ‘Marquette’ pomace by 48%. However, this reduction was not significantly different from that caused by HA drying (53%). The reason for higher phenolics content in IR‐dried ‘Edelweiss’ pomace can be the improved extraction of phenolic compounds through higher intermolecular interaction. Also, the interconversion of phenolic compounds may be accelerated by IR drying, causing an undetected compound to transform into a detectable compound (Sui et al., 2014).

### 3.4 Antioxidant activity

**FIGURE 3:**
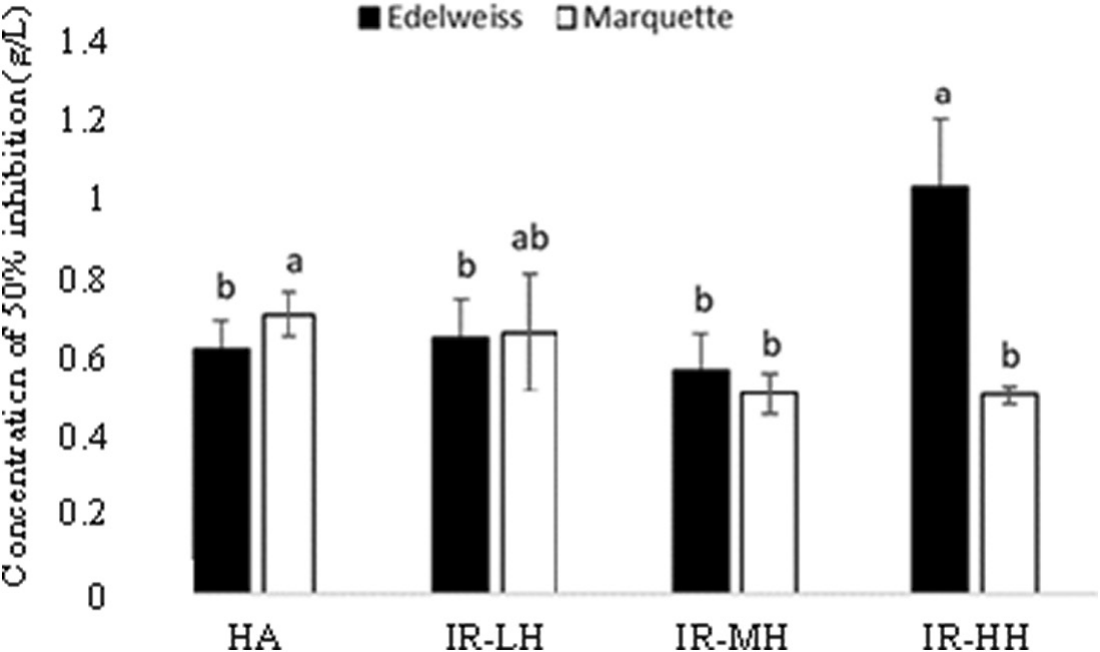
Antioxidant activity of Edelweiss (black bars) and Marquette (white bars) dry pomace extracts. Expressed as a concentration in g/L of extract needed to reach 50% DPPH inhibition. HA, Hot air drying; IR‐HH, Infrared high heating; IR‐LH, Infrared low heating; IR‐MH, Infrared modified heating.

A significantly lower antioxidant activity was observed in ‘Edelweiss’ pomaces dried with IR‐HH than dried with HA, as the concentration of ‘Edelweiss’ pomace dried with IR‐HH was higher than HA to inhibit 50% of DPPH (Figure 3).

On the other hand, the antioxidant activity was higher in ‘Marquette’ pomace after IR‐MH and IR‐HH drying than dried with HA. The increase in antioxidant activity could be explained by more accessibility of phytochemical compounds from the cell matrix and higher radical‐scavenging activity (Gahler et al., 2003). There are controversial reports regarding the impact of thermal treatment on the antioxidant activity of GP. For instance, Carmona‐Jiménez et al. (2018) reported a 2.4 and 2.8 times higher antioxidant activity for GP dried at 40°C than raw GP, correlating to the higher phenolic contents in the dried samples (*r* = .66). A decrease of 28% and 50% in the antioxidant activity of GP peel was caused by drying temperatures at 100°C and 140°C, respectively, using an air‐circulating oven (Larrauri et al., 1997). On the contrary, thermal furnace and autoclave treatment at 100°C did not change the antioxidant activity of grape seed extract and GP (Chamorro et al., 2011).

### 3.5 Microbiological quality

**FIGURE 4:**
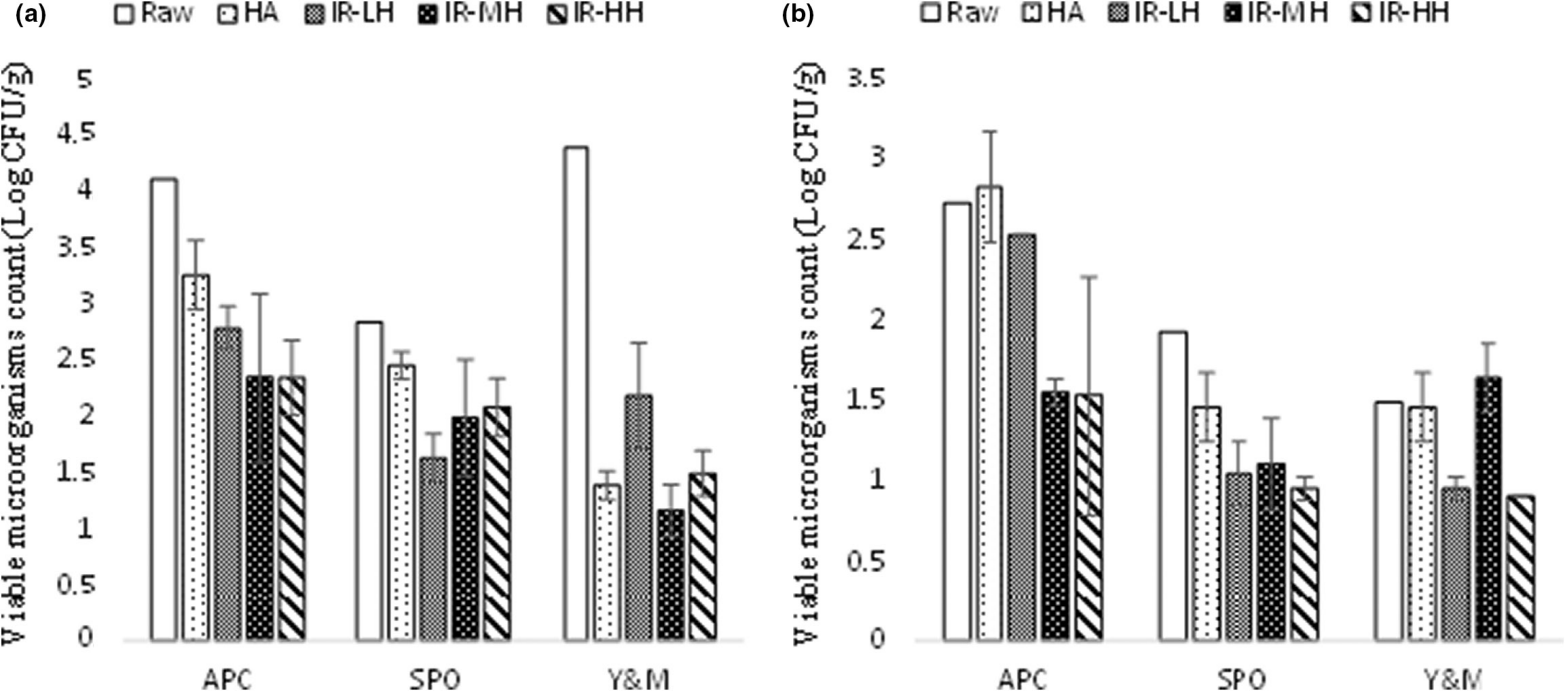
Microbial properties of Edelweiss (a) and Marquette (b) dry pomaces. APC, Aerobic plate count; HA, Hot air drying; IR‐HH, Infrared high heating; IR‐LH, Infrared low heating; IR‐MH, Infrared modified heating; SPO, Spore‐forming bacteria (aerobic); Y&M, Yeast and mold.

The IR and HA drying methods used in the current study reduced the microbial count present on the GP. The IR heating treatments caused significant decontamination of microorganisms, particularly APC, SPO, and Y&M, in the Edelweiss pomaces (Figure 4a) and SPO in the ‘Marquette’ pomaces (Figure 4b).

The most significant reduction was observed for Y&M on the dried ‘Edelweiss’ pomaces with HA and IR heating treatments. The IR drying caused an average decrease of about 99.9% Y&M after drying Edelweiss pomace. For ‘Marquette’, microorganisms were significantly reduced for IR‐HH‐ and IR‐LH‐dried samples considering all three microorganism classes studied here. Also, the IR‐MH considerably reduced APC and SPO contents in ‘Marquette’ pomaces. The microbial inactivation using HA was generally higher for ‘Edelweiss’ pomaces but lower for ‘Marquette’ pomaces. The Y&M population has shown sensitivity to heat, as heating the raw GP up to 50°C and adding 45 mg/L total SO_2_ reduced the yeast population (*Brettanomyces bruxellensis*). A 1–2 log reduction was seen after heating for 1–5 min (Cartwright et al., 2018).

Similarly, IR heating and convective heating at 80°C and 90°C reduced significant amounts of micro‐organisms, including Y&M and bacteria on GP. In that study, IR heating was applied for 14 min to kill yeasts (Sui et al., 2014). In the current study, the significantly higher reduction in APC caused by IR‐MH and IR‐HH than IR‐LH could be attributed to the higher temperature received by the product surface. The IR treatment appeared more advantageous than convective heating in killing heat‐resistant micro‐organisms caused by penetrating cells and damaging the protein inside (Sui et al., 2014).

Other irradiation treatments rather than IR heating have also shown microbial inactivation. For example, gamma irradiation at 6 kGy inhibited bacteria and Y&M growth during the first 3 and 4 days after storage at 24°C, respectively, suggesting that treated GP can be stored for a longer time (Ayed et al., 1999).

Further work is needed and currently focuses on evaluating the chemical and microbial stability of flours obtained from IR‐treated GP under various storage conditions.

## 4 CONCLUSION

The effect of IR heating on the valorization of cold‐hardy GP in terms of drying and stabilizing the wine GP was investigated. The effectiveness of IR drying was compared with conventional HA drying. The physicochemical properties, including color, phenolic degradation, and microbial inhibition, were evaluated. All the IR heating treatments could reduce the grape MC in a significantly shorter time than HA. The best IR drying parameters were found to be IR‐MH, which resulted in the fastest drying of GP. The HA and IR heating did not cause any significant differences in color and phenolic degradation. However, the drying method impacted the antioxidant activity depending on the type of grape, which could be attributed to the presence of other antioxidants. On an average, a higher microbial inactivation was achieved when the GP was treated with IR heating. Overall, IR heating, mainly IR‐MH, was the most suitable processing treatment for GP with shorter drying time, high quality, and safety efficiencies. Establishing a rapid and effective drying approach is essential because of the lengthier drying times and losses in polyphenolic content associated with traditional drying processes.

## FUNDING INFORMATION

This study was supported by the U.S. Department of Agriculture's (USDA) Agricultural Marketing Service through grant SCBG‐1427‐4. Its contents are solely the authors' responsibility and do not necessarily represent the official views of the USDA.

## CONFLICT OF INTEREST

The authors declare that there is no conflict of interest.

## Data Availability

Research data are not shared.
